# Effects on Gene Transcription Profile and Fatty Acid Composition by Genetic Modification of Mevalonate Diphosphate Decarboxylase MVD/Erg19 in *Aspergillus Oryzae*

**DOI:** 10.3390/microorganisms7090342

**Published:** 2019-09-11

**Authors:** Zhihong Hu, Hui Huang, Yunlong Sun, Yali Niu, Wangzishuai Xu, Qicong Liu, Zhe Zhang, Chunmiao Jiang, Yongkai Li, Bin Zeng

**Affiliations:** Jiangxi Key Laboratory of Bioprocess Engineering and Co-Innovation Center for In-vitro Diagnostic Reagents and Devices of Jiangxi Province, College of Life Sciences, Jiangxi Science & Technology Normal University, Nanchang 330013, Jiangxi, China; huzhihong426@163.com (Z.H.); hhuang2576681471@163.com (H.H.); ylsun0558@126.com (Y.S.); niuyali2012@163.com (Y.N.); xuwang960124@163.com (W.X.); lqc9762@163.com (Q.L.); zz2007138221@163.com (Z.Z.); jiangcm0810@163.com (C.J.); lyk2008china@163.com (Y.L.)

**Keywords:** mevalonate diphosphate decarboxylase, *Aspergillus oryzae*, transcriptome, fatty acid, ergosterol biosynthesis

## Abstract

Mevalonate diphosphate decarboxylase MVD/Erg19 is required for ergosterol biosynthesis, growth, sporulation, and stress tolerance in *Aspergillus oryzae*. In this study, RNA-seq was used to analyze the gene transcription profile in *AoErg19* overexpression (OE) and RNAi strains. There were 256 and 74 differentially expressed genes (DEGs) in *AoErg19* OE and RNAi strains, respectively, compared with the control strain (CK). The most common DEGs were transport- and metabolism-related genes. Only 22 DEGs were obtained that were regulated in both OE and RNAi strains. The transcriptomic comparison between CK and *AoErg19* overexpression strain (CK vs. OE), and between CK and *AoErg19* RNAi strain (CK vs. RNAi) revealed that the greatest difference existed in the number of genes belonging to the cytochrome P450 family; 12 were found in CK vs. OE, whereas 1 was found in CK vs. RNAi. The expression patterns of lipid biosynthesis and metabolism related genes were altered in OE and RNAi strains, either by gene induction or suppression. Moreover, the total fatty acid content in the RNAi strain was 12.1% greater than the control strain, but no difference in total acid content was found between the overexpression strain and the control strain. Therefore, this study highlights the gene expression regulation within mevalonate (MVA), ergosterol biosynthesis, and fatty acid biosynthesis pathways.

## 1. Introduction

The mevalonate (MVA) pathway is conserved among organisms [1]. This pathway produces isoprenoids, the most numerous and structurally diverse family of natural compounds [2]. Terpenoids, a class of isoprenoids, can be used commercially as flavor and fragrance compounds and as antimalarial or anticancer drugs [3]. Isoprenoids can be used as an important component of synthetic rubber and high-energy biofuel [4,5]. The MVA pathway is initiated by the condensation of two acetyl-CoA molecules, which is catalyzed by acetoacetyl-CoA thiolase (EC 2.3.1.9), forming acetoacetyl-CoA [6]. A third acetyl-CoA then reacts with acetoacetyl-CoA to form 3-hydroxy-3-methylglutaryl-CoA (HMG-CoA) via hydroxymethylglutaryl-CoA synthase (EC 2.3.3.10) [4]. HMG-CoA is further reduced by HMG-CoA reductase (EC 1.1.1.34), forming mevalonate [7]. Mevalonate is then pyrophosphorylated by mevalonate kinase (EC. 2.7.1.36) and phosphomevalonate kinase (EC 2.7.4.2) successively, producing mevalonate 5-diphosphate (MVA-PP). Finally, mevalonate diphosphate decarboxylase (MVD; EC 4.1.1.33) catalyzes the decarboxylation of six-carbon MVA-PP to five-carbon isopentenyl diphosphate (IPP) [8], which the basic structure required for the biosynthesis of isoprenoids, and acts as an important cellular intermediate [9]. 

In fungi, IPP is a precursor required for ergosterol biosynthesis. Ergosterol is a fungal cell membrane component that is important for cell membrane fluidity and permeability and also plays important roles in fungal growth, reproduction, and stress tolerance [10]. Therefore, the *MVD* gene is also known as *erg19* in fungi. In higher plants, the IPP molecule acts as a basic skeleton for plant-specific molecule biosynthesis, including growth regulators (e.g., gibberellins and abscisic acid), photosynthetic pigments, phytotoxins, phytoalexins, other plant defense compounds, aromatic terpenoids, and natural rubbers [11,12]. In animals, IPP is the precursor for cholesterol, dolichol, haem A, and ubiquinone biosynthesis, which are involved in membrane biogenesis, glycoprotein synthesis, and electron transport [13]. Thus, MVD is a key enzyme of the MVA pathway [8] and plays important roles in organisms such as microbes, plants, and animals [14].

As mentioned, the MVA pathway is initiated by the condensation of two acetyl-CoA molecules [6]. In cells, acetyl-CoA is generated from pyruvate metabolism or fatty acid beta-oxidation [15]. Acetyl-CoA is also a substrate for fatty acid biosynthesis or elongation [16]. Cellular lipid metabolism is a complex network of pathways that is precisely regulated and coordinated to maintain lipid homeostasis [3,17]. Therefore, MVA pathway and fatty acid biosynthesis may have a closely regulated network. For example, in keratinocytes, HMG-CoA reductase inhibition (part of the MVA pathway) increases fatty acid synthesis [18]; proteomic analysis has revealed that fatty acid synthesis and mevalonate pathways play a critical role in the survival of pancreatic cancer stem cells [19]; the fatty acid decarboxylase of *Jeotgalicoccus* species can also catalyze the conversion of mevalonate into 3-methyl-3-butan-1-ol [20].

Little research exists on the relationship between the MVA pathway and fatty acid biosynthesis in *Aspergillus oryzae*, which is one of the most industrially important filamentous fungi. *A. oryzae* is a FDA and WHO identified safe production filamentous fungus that has been used in the manufacturing of oriental fermented foods such as sauce, miso, and sake for thousands of years. It is also commercially used for valuable enzyme production. Therefore, more and more studies focused on the molecular and cellular biology of *A. oryzae* [21]. In our previous study, the expression pattern and subcellular location of the *AoErg19* was described in *A. oryzae*, and it was revealed that *AoErg19* is required for ergosterol biosynthesis, growth, sporulation, and stress tolerance in *A. oryzae* [22]. In the present study, RNA-seq was used to analyze gene transcription profiles of *AoErg19* overexpression and RNAi strains to identify genes that are affected by *AoErg19* expression changes, and also to identify their potential effects on lipid biosynthesis and metabolism. Moreover, the total fatty acid content in the RNAi strain was 12.1% greater than the control strain, but no difference was found between the overexpression strain vs. the control strain. Therefore, this study aims to identify details on gene expression regulation within the MVA, ergosterol biosynthesis, and fatty acid biosynthesis pathways, which may lay the foundation for the genetic engineering of lipid biosynthesis and other metabolic pathways in this industrially important fungus.

## 2. Results

### 2.1. Phenotypic Characterization of A. oryzae AoErg19 Overexpression and RNAi Strains 

**Figure 1:**
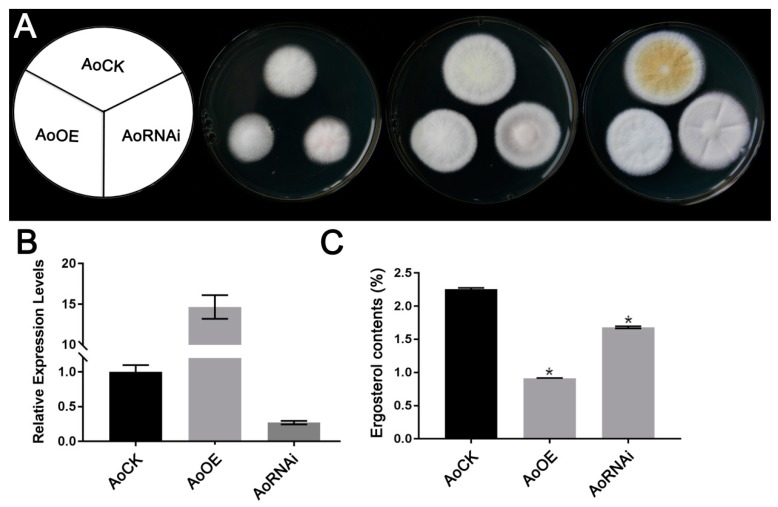
Colony morphologies, *AoErg19* expression level, and ergosterol contents in control strain (CK), overexpression (OE), and RNAi strains. (**A**) Colony morphologies of CK, OE, and RNAi strains on DPY (dextrin-peptone-yeast extract) medium after 48 h, 60 h, or 72 h of incubation. From left to right: scheme showing different transgenic strains, 48 h, 60 h, and 72 h colony morphologies. (**B**) The relative expression level of *AoErg19* in CK, OE, and RNAi strains. (**C**) Ergosterol content of CK, OE, and RNAi strains. In B and C, values are given as the mean of three replicates plus SE, *p < 0.05.

Both OE and RNAi strains showed delayed growth compared with the control (Figure 1A). In order to get rid of the DEGs caused by different growth stages, we selected 48 h mycelium for RNAseq analysis. The *AoErg19* expression level and ergosterol contents of the selected genotype strains are shown in Figure 1B,C.

### 2.2. Transcriptome Overview

The numbers of total reads for all strains were greater than 38,391,610. Among those reads, the mapped reads ranged from 86.95% to 91.69%, and more than 87% were unique mapped reads. The %≥Q30 was greater than 91% and the GC content for all treatments was approximately 52%. The Pearson’s correlation coefficient between two repeats of each strain was greater than 0.767, which indicates that the RNA-seq results of each strain were repeatable and reliable. A summary of the RNA-seq results is shown in Table 1. 

### 2.3. Differentially Expressed Genes in OE and RNAi Strains

**Figure 2:**
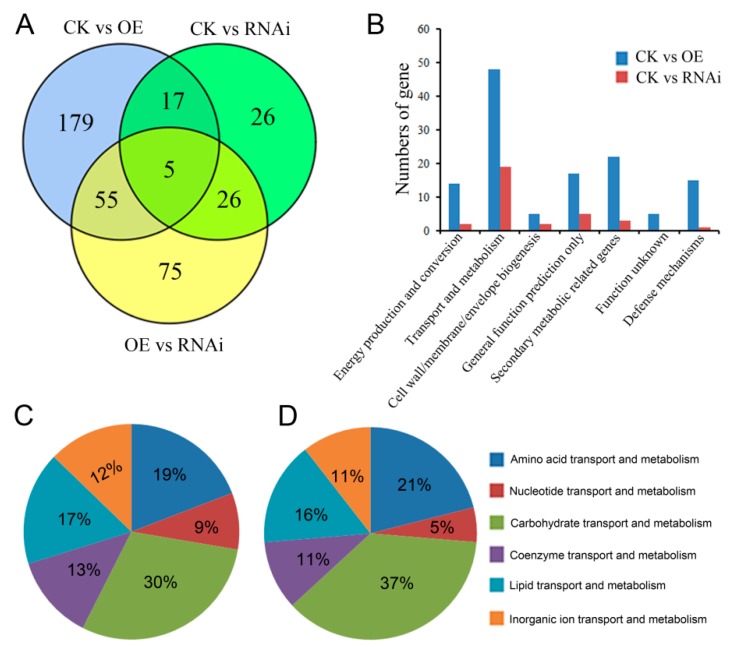
Distribution and classification of DEGs in CK vs. OE, CK vs. RNAi, and OE vs. RNAi. (**A**) Venn diagram displaying the DEG distribution of samples. (**B**) COG function classification of DEGs in samples. (**C**,**D**) The classification of DEGs involved in transport and metabolism for CK vs OE (**C**) and CK vs RNAi (**D**).

The number of DEGs for CK vs. OE, CK vs. RNAi, and OE vs. RNAi were 256, 74, and 161, respectively. A summary of the DEGs is shown in Table 2. All DEGs for CK vs. OE, CK vs. RNAi, and OE vs. RNAi are shown in Tables S1–S3. For these DEGs, only five DEGs were commonly shared among the three groups, and the other commonly shared DEGs between every two groups were 22, 31, and 60, respectively (Figure 2A). The transcription levels of *AoErg19* in RNA-seq results confirmed that *AoErg19* was overexpressed and knocked down in OE and RNAi strains, respectively, compared with CK (Figure S1), which demonstrate the reliability of the RNA-seq results. DEGs were analyzed by Cluster of Orthologous Groups of proteins (COG) functional classification. The COGs mapped to DEG gene function were divided into seven classes: energy production and conversion, transport and metabolism, cell wall/membrane/envelope biogenesis, general function prediction only, secondary metabolic-related genes, function unknown, and defense mechanisms (Figure 2B). Our previous studies have revealed that the most DEGs associated with ergosterol biosynthesis inhibitors are transport- and metabolism-related genes [23]. Consistent with these results, in the present study, the most common DEGs were also transport- and metabolism-related genes, which accounted for 38.1% and 59.4% of the DEGs for CK vs. OE and CK vs. RNAi, respectively. These transport- and metabolism-related genes were divided into six classes (Figure 2). Among these six classes, the most common for both CK vs. OE and CK vs. RNAi are carbohydrate transporters (Figure 2C,D).

### 2.4. Comparison of Gene Expression Profiles in OE and RNAi Strains

Results show that there are 22 common DEGs (Table 3). Except for EIT82403 and EIT77689, the most common DEGs showed the same trends in CK vs. OE and CK vs. RNAi. Most of the common DEGs were associated with cell membrane proteins or transporters, accounting for 41%; the second were associated with energy production and conversion, and transcription. There were only five commonly shared DEGs among the three groups: EIT76067, EIT82403, EIT76073, EIT77689, and EIT79995.1, which are shown in bold in Table 3. The details regarding expression differences and function of these genes are shown in Table 3.

### 2.5. Expression of Cytochrome P450 Family Genes in the Over-Expression Strain 

By analyzing the different DEGs in OE and RNAi strains, it was discovered that major differences could be classified into secondary metabolic-related genes, which can be seen in Figure 2B. There were 22 and three DEGs associated with secondary metabolism in CK vs. OE and CK vs. RNAi, respectively. A detailed analysis of these genes revealed that 12 DEGs (55%) belonged to the cytochrome P450 family in CK vs. OE; however, only one DEG belonged to the cytochrome P450 family for CK vs. RNAi. Fourteen DEGs belonged to the cytochrome P450 family for OE vs. RNAi. The name and expression level of these genes are shown in Table 4. 

### 2.6. Expression Pattern of Genes Involved Lipid Biosynthesis and Metabolism 

**Figure 3:**
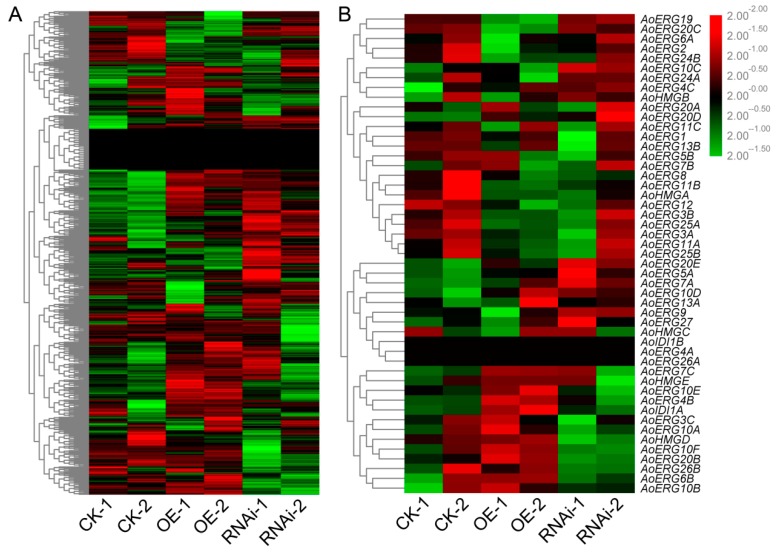
The expression profiles of lipid biosynthesis and metabolism, and ergosterol biosynthesis genes. (**A**) Expression of genes involved in lipid biosynthesis and metabolism. (**B**) Expression of genes involved in ergosterol biosynthesis.

**Figure 4:**
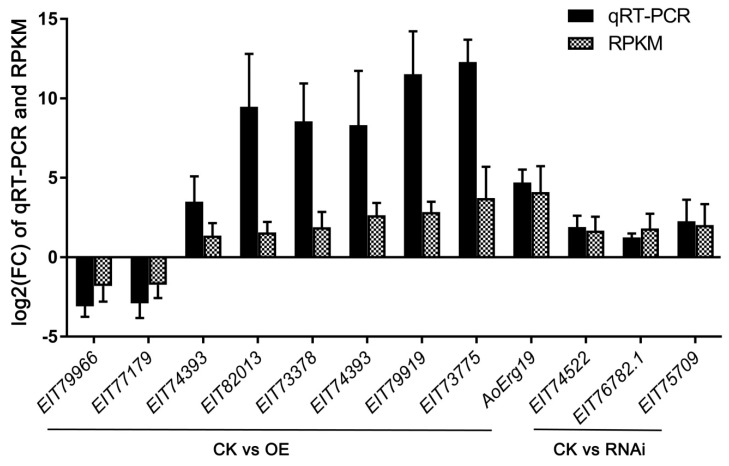
The expression level of DEGs involved lipid and ergosterol biosynthesis and transportation confirmed by qRT-PCR. Values are given as the mean of three replicates plus SE.

A heatmap of the expression patterns of these lipid-related genes revealed that almost all of the genes displayed altered expression patterns, either via induction or suppression, in the OE and RNAi strains (Figure 3A). Then, we analyzed the expression patterns of ergosterol biosynthesis genes and found that most of these genes also displayed altered expression patterns (Figure 3B). The DEGs involved in lipid biosynthesis and metabolism were also analyzed. As shown in Table 3, there were nine DEGs found in CK vs. OE, including five ergosterol biosynthesis genes; only three DEGs were found in CK vs. RNAi. Among all the DEGs involved in lipid biosynthesis and metabolism, only two genes (*EIT79966* and *EIT77179*) were down-regulated; the others were up-regulated. The detailed functions of these genes are shown in Table 5. Besides, qRT-PCR was performed to confirm the reliability and availability of the DEGs involved lipid and ergosterol biosynthesis and transportation. The results of qRT-PCR correlated with that of transcriptome analysis (Figure 4).

### 2.7. Fatty Acid Composition in Over Expression and RNAi A. oryzae Strains

As the initial substrates for ergosterol and fatty acid biosynthesis pathways are acetyl-CoA. Our previous study showed that ergosterol contents were less in OE and RNAi strains compared to the control strain [22]. Thus, we analyzed whether the fatty acid compositions were also affected in OE and RNAi strains. Results showed that in OE strains, the compositions of fatty acid were changed, for example C14:0, C16:1, C18:0, C18:1n9c, and C24:0 increased a little, but the contents of total fatty acid, unsaturated fatty acid (UFA) and saturated fatty acid (FA) showed no significant change compared with CK strain (Table 6). However, in the RNAi strain, both the fatty acid compositions and contents showed differences compared with CK strain. In RNAi strains, the total fatty acid content was 12.1% greater than that of the control strain, which is approximately equal to the differences in UFA (12.0%) and FA (12.4%) content. Therefore, the total fatty acid content in the RNAi strain increased, while no difference in total acid content was found between the overexpression strain and the control strain.

## 3. Discussion 

Cellular lipid metabolism involves a complex network of pathways, and is precisely regulated and coordinated to maintain lipid homeostasis [3,17]. Our previous study showed that both the overexpression and RNAi of *AoErg19*, one of the key genes in MVA pathway, were associated with lower ergosterol content in *A. oryzae*. In this study, we highlighted the transcription profile changes that occur in *AoErg19* overexpression and RNAi strains. The fatty acid contents of these strains were also analyzed. This study lays a foundation for understanding the gene expression regulation along the MVA, ergosterol biosynthesis, and fatty acid biosynthesis pathways.

### 3.1. Effects of AoErg19 on Gene Transcription in A. oryzae 

We identified 256 and 74 DEGs for CK vs. OE and CK vs. RNAi, respectively. The number of DEGs appears to be smaller compared with other transcriptome analyses, indicating that the effect on the gene transcription profiles by modification of *AoErg19* is specific. Thus, these DEGs are likely to be genes that are regulated directly by *AoErg19*. The number of DEGs found in CK vs. OE was far greater than the number in CK vs. RNAi, which can be explained by the differences in phenotype, mRNA levels, and ergosterol content of the OE strain, which were more pronounced than those of RNAi strains [22]. Former transcriptome analysis revealed that the most common DEGs associated with the inhibition of ergosterol biosynthesis were transport- and metabolism- related genes [23,24]. Consistent with these results, the most common DEGs for CK vs. OE and CK vs. RNAi were also transport- and metabolism-related genes, which may be caused by the changes in ergosterol content, as this is a component of cell membranes, and most transporters are located within the cell membrane. We also compared transport- and metabolism-related genes with those identified from ergosterol biosynthesis inhibitor treatment and found that there were no common genes. This indicates that different treatment methods or transgenic strains resulted in different changes in cell membrane components, resulting in differences in transporter gene expression. Our former studies revealed that the OE and RNAi strains had defects in growth and sporulation [22]. However, little DEGs involved in growth and sporulation were identified. This may because of that the RNA-seq analysis was performed in 48-h-old strains, the growth differences between transgenic strains and CK is not obvious and sporulation usually happens after 48-h cultivation.

### 3.2. Comparison of Gene Expression Profiles in OE and RNAi Strains

A comparison of DEGs for CK vs. OE and CK vs. RNAi revealed that there were 22 genes affected by both overexpression and RNAi of *AoErg19*, and only five DEGs were shared among CK vs. OE, CK vs. RNAi, and OE vs. RNAi. Most of the DEGs found were associated with cell membrane proteins or transporters. The second most common were associated with energy production and conversion, and transcription related genes. The changes in energy production and conversion-related genes can be explained by how altered ergosterol content impairs mitochondrial structure [25,26,27]. In investigating the differences of DEGs between CK vs. OE and CK vs. RNAi, a large number of cytochrome P450 family genes were found. For example, there were 12 DEGs associated with the cytochrome P450 family in CK vs. OE, but only 1 was found for CK vs. RNAi. This can be explained by how in the OE strain, the intermediates IPP, dimethylallyl-PP, geranyl diphosphate, or farnesyl-PP could have accumulated, which can be toxic to cells. Therefore, cytochrome P450 family genes may have been regulated to metabolize these intermediates.

### 3.3. Expression Pattern of Lipid Biosynthesis and Metabolism Genes, and Fatty Acid Content in OE and RNAi Strains 

**Figure 5:**
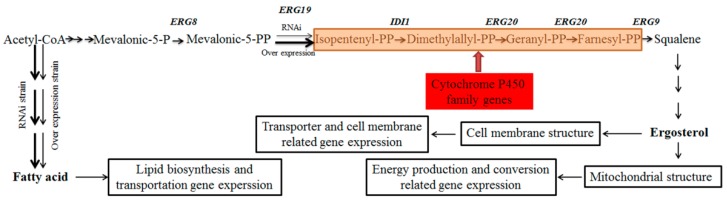
Model for genetic modification *AoErg19* mRNAi level affecting the gene transcription profile and fatty acid composition in *A. Oryzae*. In the *AoErg19* over expression strain, more acetyl-CoA was consumed by the MVA pathway, and IPP, dimethylallyl-PP, geranyl diphosphate, or farnesyl-PP accumulated as a result. Cytochrome P450 family genes were activated to metabolize these intermediates. In the RNAi strain, more acetyl-CoA was consumed by fatty biosynthesis. Both OE and RNAi strains showed differences in ergosterol contents, which can affect the cell membrane structure and mitochondrial structure, further affecting transport and cell membrane related gene expression and energy production and conversion related gene expression. Meanwhile, changes in fatty acid in cell resulted in the altered expression of lipid biosynthesis and transportation related genes. The bold arrowhead indicates that more substrate enters into the pathway.

Although expression patterns of almost all genes related to lipid biosynthesis and metabolism displayed altered expression patterns (Figure 3A), only nine DEGs for CK vs. OE (including five ergosterol biosynthesis genes) and three DEGs for CK vs. RNAi, were identified. Fatty acid composition analysis showed that although compositions of RNAi and OE fatty acids were different to those of the control, the contents of total fatty acid, UFA, and FA were only increased in RNAi strains. A possible reason is that reduction of *AoErg19* mRNAi levels may have led to a decrease in the acetyl-CoA available for the biosynthesis of ergosterol. Therefore, more acetyl-CoA may have been used for fatty acid biosynthesis. Few DEGs involved in lipid and transportation were identified in CK vs. RNAi, possibly because of acetyl-CoA in fatty acid biosynthesis boosting the overall fatty acid biosynthesis pathway with minimal involvement of specific genes; altered expression levels may have been too insignificant to be detected. This is consistent with the result that the total fatty acid difference was approximately equal to that of UFA (12.0%) and FA in RNAi strains. A model for genetic modification of *AoErg19* mRNAi levels affecting gene transcription profiles and fatty acid composition in *A. Oryzae* is shown in Figure 5. 

## 4. Method and Materials

### 4.1. Strain and Culture Conditions

*A. oryzae* 3.042 (CICC 40092) was obtained from the China Center of Industry Culture Collection (Beijing, China) and used as the wild type strain. *A. oryzae* 3.042 △*pyrG* was constructed by our laboratory [28] and used as the background for over expression and RNAi of *AoErg19*. *A. oryzae* was cultured on (Czapek-Dox Agar) medium (2% sucrose, 0.2% NaNO_3_, 0.1% KH_2_PO_4_, 0.05% MgSO_4_, 0.05% KCl, 0.05% NaCl, 0.002% FeSO_4_, 1.6% agar, pH 5.5) at 30 °C. 

### 4.2. Preparation of cDNA Libraries and RNA Sequencing

Approximately 0.5 g mycelia after 48 h cultivation were used for RNA sequencing. Total RNA isolation was performed using a fungal RNA kit (Omega Bio-tek, Norcross, GA, USA) according to the manufacturer’s instructions, with the addition of RNase-free DNase I treatment (Omega). RNA concentration and integrity were analyzed using a NanoDrop ND-1000 spectrophotometer (Thermo Scientific, Wilmington, DE, USA), and using a Bioanalyzer 2100 (Agilent Technologies, Palo Alto, CA, USA). Equal quantities of RNA of each pool from three individual cultures were used for cDNA library construction to ensure reliability and reproducibility. The total RNA was then enriched using oligo (dT) magnetic beads. After enrichment, mRNA was digested into short pieces in fragmentation buffer at 94 °C for 5 min. Then, mRNA fragments were used as templates to synthesize first-strand cDNA using random hexamer-primers. Second-strand cDNA was synthesized using DNA Polymerase I and RNase H. Following end-repair and adaptor ligation, the products were amplified by PCR and purified with a QIAquick PCR purification kit (Qiagen, Beijing, China) to create a cDNA library. Finally, the constructed libraries were sequenced by Biomarker Technologies Co. using an Illumina HiSeq 2500 platform (Illumina, San Diego, CA, USA) to generate 125 bp paired-end reads. Two biological replicates for RNA seq were used for each strain. The sequencing data were deposited in the NCBI/SRA database (Bioproject: PRJNA558619; BioSample: SAMN12496167).

### 4.3. Mapping Reads to the A. oryzae Reference Genome and Normalized Gene Expressions

Low-quality reads, adaptor sequences and reads with N’s (uncertain bases) were removed to obtain high quality clean-read datasets, which were aligned to the *A. oryzae* genome (https://www.ncbi.nlm.nih.gov/genome/526?genome_assembly_id=29881) using Tophat (v2.0.7) software. The Q20, Q30, GC-content and sequence duplication rate of the clean data were calculated. Transcript abundances were normalized by the reads per kilobase per million mapped reads (RPKM) metric to identify differentially expressed genes (DEGs). The false discovery rate (FDR) within 0.05 and log_2_ (fold change) over 1 were set as the threshold for identification DEGs between samples [29,30,31]. 

### 4.4. Quantitative Real-time Reverse Transcription–PCR

Total RNA was isolated as described above. The cDNAs were synthesized from 1 μg of total RNA using the Prime Script™ RT Reagent Kit (Perfect Real Time; Shiga, Takara). All of the quantitative real-time reverse transcription–PCR (qRT–PCR) measurements were performed using a CFX96 Real-Time PCR Detection System (Bio-Rad, Hercules, CA, USA) with TB-Green Premix Ex Taq (Takara, Shiga, Japan), according to the manufacturer’s instructions. The housekeeping gene *GAPDH* was used as a normalization control. The relative expression was calculated by using the formula 2^–ΔΔCt^. All the experiments were performed for each biological replicate. The primer sequences for qRT–PCR are provided in Table S5.

### 4.5. Measurement of Fatty Acid Contents

Total lipids extraction was performed according to a previously described method [32,33]. Briefly, 48-h-old *A. oryzae* mycelia were collected and vacuum freeze-dried to a constant weight. Then, the mycelia were pulverized, weighed, and placed in a 50-mL Falcon tube for lipid extraction. Subsequently, 2.3 mL of dimethyl sulfoxide, *p*-hydroxymercuribenzoic acid (1 mmol/L final concentration), and 2,6-di-*tert*-butyl-*p*-cresol (7 mmol/L final concentration) were added. The suspension was maintained in a water bath at 100 °C for 1 h and then cooled at room temperature. Then, 13.3 mL of 1:1 chloroform–methanol was added and the mixture was gently shaken for 3 min then maintained at 4 °C overnight. To this, 6.7 mL of chloroform was added and 2.5 mL water was introduced and the tube was gently shaken for 3 min; after centrifugation at 6000 rpm for 10 min, the aqueous phase was eliminated and the organic phase washed 3× with 1.6 mL of 2 mol/L KCl·H2O. Finally, the lipid extract was then dried in a rotavapor and dissolved in 5 mL of chloroform. The lipid extracts were incubated in anhydrous methanol with 2% H_2_SO_4_ at 70 °C for 2 h to obtain fatty acid methyl esters (FAMEs). The FAME components were extracted by *N*-hexane, methyl salicylate as internal standard. A mixed fatty acid methyl ester standard (Nu-Chek Prep Inc. Elysian, MN, USA) was used to draw standard curve. Samples were separated and analyzed using a GC-MSD (Agilent 7890A/5975C Gas-Mass Spectromete) equipped with an Agilent DB-WAX capillary polar column (30 m × 0.25 mm ID × 0.25 μm). MSD ChemStation software was used to extract the peak area and retention time. Fatty acid contents were calculated using the standard curve and are expressed as mg/g dry weight cells.

### 4.6. Data Analysis

All of the data in this study are presented as mean ± SE of three replicates. Significant differences were determined through a one-tailed Student’s t-test or one-way analysis of variance (ANOVA) followed by Tukey’s honestly significance difference (HSD) test for mean comparison.

## 5. Conclusions

To conclude, this study have investagited the gene transcription profile in *AoErg19* OE and RNAi strains of *A. oryzae*. The comparison of the DEGs in CK vs OE and CK vs RNAi revealed that the most common DEGs were transport- and metabolism-related genes and the greatest difference existed in the number of genes belonging to the cytochrome P450 family. Moreover, the total fatty acid content in the RNAi strain was 12.1% greater than the control strain, but no difference in total acid content was found between the overexpression strain and the control strain. 

## Figures and Tables

**Table 1:** Summary of RNA-seq results.

Tag Classification	WT		Over Expression		RNAi	
	1	2	1	2	1	2
Total reads	55,002,642	42,717,586	41,217,752	47,194,454	38,391,610	41,080,464
Percentage of mapped reads	89.33%	91.69%	88.29%	87.58%	86.95%	87.83%
Percentage of unique reads	89.04%	91.38%	88.02%	87.27%	87.27%	87.51%
%≥Q30	91.75%	92.19%	92.17%	91.96%	91.61%	92.59%
GC content	52.58%	52.58%	52.86%	52.75%	52.78%	52.75%
Pearson’s correlation coefficient	0.919		0.98		0.767	

**Table 2:** Summary of the differentially expressed genes (DEG) in OE and RNAi strains.

DEG Set	DEG Number	Up-Regulated	Down-Regulated
CK vs. OE	256	148	108
CK vs. RNAi	74	41	33
OE vs. RNAi	161	87	74

**Table 3:** Genes regulated in both over expression and RNAi strains.

Accession Number	Putative Production Encoded by the Gene	Fold Changes	
		OE	RNAi
**Cell Membrane Protein or Transporter**			
EIT81161	Integral component of membrane, function unknown	−3.14	−2.95
EIT72587	Ca^2+^ transporting ATPase	−1.63	−1.45
EIT83492	Transmembrane amino acid transporter protein	4.48	4.67
**EIT76067**	Inorganic ion transport and metabolism	4.48	1.87
**EIT82403**	Integral component of membrane	2.55	−4.15
EIT83199	Cellular membrane protein with unknown function	2.19	2.79
EIT79933	Acyl-CoA hydrolase activity	−7.71	−7.57
EIT81552	Phosphoribosylglycinamide synthetase	1.59	1.53
**Energy production and conversion**			
EIT72870	Mitochondrial protein FMP32	2.39	1.93
EIT80752	FAD-linked oxidoreductase	−4.30	−4.44
EIT83112	FAD-dependent oxidoreductase	-Inf	-Inf
**Transcription**			
EIT75959	Demethylsterigmatocystin 6-O-methyltransferase	3.48	5.32
EIT77884	RNA polymerase II transcription factor activity	−7.44	−7.00
EIT77860.1	Transcriptional adapter 2	−5.52	−8.46
**Defense mechanisms**			
**EIT76073**	Beta-lactamase class C	5.30	1.81
**EIT77689**	Fungal fucose-specific lectin	−1.47	2.13
**Unknown function and others**			
EIT81216	Hypothetical protein	−2.98	−2.13
EIT81230	Hypothetical protein	−2.19	−1.98
EIT74353.1	Hypothetical protein	2.98	7.14
EIT72406	Hypothetical protein	−1.64	−1.32
EIT77940	Tetratricopeptide repeat protein	−1.54	−1.58
**EIT79995.1**	Alkyl sulfatase	4.59	2.88

–inf means minus infinity.

**Table 4:** The DEGs of cytochrome P450 family.

Accession Number	CK vs. OE	CK vs. RNAi	OE vs. RNAi
EIT79521	−3.76	ND	ND
EIT79561	−3.35	ND	ND
EIT79542	−3.25	ND	ND
EIT79575	−2.29	ND	ND
EIT81599	1.09	ND	ND
EIT80587	1.40	ND	−2.48
EIT73378	1.89	ND	ND
EIT82715	2.32	ND	ND
EIT83385	2.46	ND	−2.35
EIT82718	2.51	ND	−1.53
EIT81977	2.64	ND	−3.28
EIT81654	3.91	ND	−2.91
EIT80982	ND	ND	1.45
EIT73302	ND	ND	6.47
EIT81011	ND	ND	1.39
EIT75636	ND	ND	−2.27
EIT81129	ND	1.58	1.18
EIT80973	ND	ND	1.45
EIT81014	ND	ND	1.43
EIT80470	ND	ND	1.58
EIT82011	ND	ND	3.48

ND represents not detected.

**Table 5:** DEGs involved lipid and ergosterol biosynthesis and transportation.

Accession Number	Putative Production Encoded by the Gene	Fold Changes
**CK vs. OE**		
EIT79966	Lanosterol synthase (*AoErg7C*)	−1.81
EIT77179	Phosphatidylserine decarboxylase activity	−1.73
EIT74393	Enoyl reductase domain of FAS1	1.36
EIT82013	Lipid transport and metabolism	1.56
EIT73378	Sterol 14-demethylase (*AoErg11B*)	1.89
EIT74393	Fatty acid synthase complex (*AoFAT1*)	2.64
EIT79919	Sterol C-5 desaturases (*AoErg3A*)	2.84
EIT73775	Oxidoreductase activity	3.72
EIT78501	Diphosphomevalonate decarboxylase (*AoErg19*)	4.10
**CK vs. RNAi**		
EIT74522	Enoyl-(Acyl carrier protein) reductase	1.67
EIT76782.1	Alpha-glucosidase	1.81
EIT75709	Phosphogluconate dehydrogenase	2.03

**Table 6:** Fatty acid composition in overexpression and RNAi strains (mg/g).

Fatty Acid	CK	OE	RNAi
C14:0	0.014 ± 0.0031	0.047 ± 0.007*	0.019 ± 0.003
C15:0	0.242 ± 0.012	0.238 ± 0.013	0.249 ± 0.038
C16:0	3.448 ± 0.810	3.523 ± 0.810	4.010 ± 0.910*
C16:1	0.061 ± 0.011	0.104 ± 0.012*	0.059 ± 0.009
C17:0	0.112 ± 0.023	0.120 ± 0.009	0.189 ± 0.031*
C18:0	0.183 ± 0.025	0.276 ± 0.0130*	0.385 ± 0.016*
C18:1n9c	2.102 ± 0.360	2.427 ± 0.190*	2.054 ± 0.540
C18:2n6c	18.834 ± 1.890	17.773 ± 1.490	22.431 ± 1.970*
C20:0	0.006 ± 0.001	0.010 ± 0.001	0.012 ± 0.021*
C20:1	0.016 ± 0.003	0.022 ± 0.002	0.018 ± 0.003
C18:3n3	1.214 ± 0.230	1.291 ± 0.190	2.015 ± 0.350*
C20:2	0.060 ± 0.011	0.059 ± 0.003	0.074 ± 0.019
C22:0	0.008 ± 0.009	0.011 ± 0.003	0.021 ± 0.002
C22:1n9	0.211 ± 0.013	0.291 ± 0.013	0.369 ± 0.018*
C20:3n3	0.019 ± 0.001	0.026 ± 0.019	0.040 ± 0.005*
C23:0	0.008 ± 0.001	0.011 ± 0.025	0.011 ± 0.004
C22:2	0.016 ± 0.003	0.017 ± 0.003	0.020 ± 0.002
C24:0	0.259 ± 0.013	0.324 ± 0.018*	0.397 ± 0.071
C24:1	0.008 ± 0.001	0.008 ± 0.001	0.009 ± 0.001
UFA	22.541 ± 2.523	22.018 ± 1.924	27.089 ± 2.916*
FA	4.280 ± 0.887	4.560 ± 0.899	5.293 ± 1.096*
Total	26.821 ± 3.410	26.578 ± 2.823	32.382 ± 4.012*

* denote significant differences compared with CK (*p* < 0.05).

## Supplementary Materials

Supplementary materials can be found at https://www.mdpi.com/2076-2607/7/9/342/s1. Table S1. DEGs for CK vs OE; Table S2. DEGs for CK vs RNAi; Table S3. DEGs for OE vs RNAi; Table S4. The primer sequences for qRT–PCR; Figure S1. The FPKM value of *AoErg19* in CK, OE and RNAi strains.

## References

[1] Hayakawa H., Sobue F., Motoyama K., Yoshimura T., Hemmi H. (2017). Identification of enzymes involved in the mevalonate pathway of Flavobacterium johnsoniae. Biochem. Biophys. Res. Commun..

[2] Martin V.J.J., Pitera D.J., Withers S.T., Newman J.D., Keasling J.D. (2003). Engineering a mevalonate pathway in Escherichia coli for production of terpenoids. Nat. Biotechnol..

[3] Liao P., Hemmerlin A., Bach T.J., Chye M.L. (2016). The potential of the mevalonate pathway for enhanced isoprenoid production. Biotechnol. Adv..

[4] Miziorko H.M. (2011). Enzymes of the mevalonate pathway of isoprenoid biosynthesis. Arch. Biochem.Biophys..

[5] Ma S.M., Garcia D.E., Reddingjohanson A.M., Friedland G.D., Chan R., Batth T.S., Haliburton J.R., Chivian D., Keasling J.D., Petzold C.J. (2011). Optimization of a heterologous mevalonate pathway through the use of variant HMG-CoA reductases. Metab. Eng..

[6] Hu Z., He B., Ma L., Sun Y., Niu Y., Zeng B. (2017). Recent Advances in Ergosterol Biosynthesis and Regulation Mechanisms in Saccharomyces cerevisiae. Indian J. Microbiol..

[7] Klug L., Daum G. (2014). Yeast lipid metabolism at a glance. FEMS yeast Res..

[8] Krepkiy D., Miziorko H.M. (2004). Identification of active site residues in mevalonate diphosphate decarboxylase: Implications for a family of phosphotransferases. Protein Sci. A Publ. Protein Soc..

[9] Bergès T., Guyonnet D., Karst F. (1997). The Saccharomyces cerevisiae mevalonate diphosphate decarboxylase is essential for viability, and a single Leu-to-Pro mutation in a conserved sequence leads to thermosensitivity. J. Bacteriol..

[10] Kodedova M., Sychrova H. (2015). Changes in the Sterol Composition of the Plasma Membrane Affect Membrane Potential, Salt Tolerance and the Activity of Multidrug Resistance Pumps in Saccharomyces cerevisiae. PLoS ONE.

[11] Zhen S., Gary R. (2012). The mevalonate pathway regulates microRNA activity in Caenorhabditis elegans. Proc. Natl. Acad. Sci. USA.

[12] Abbassi S., Patel K., Khan B., Bhosale S., Gaikwad S. (2016). Functional and conformational transitions of mevalonate diphosphate decarboxylase from Bacopa monniera. Int. J. Biol. Macromol..

[13] Goldstein J.L., Brown M.S. (1990). Regulation of the mevalonate pathway. Nature.

[14] Abbassi S.J., Vishwakarma R.K., Patel P., Kumari U., Khan B.M. (2015). Bacopa monniera recombinant mevalonate diphosphate decarboxylase: Biochemical characterization. Int. J. Biol. Macromol..

[15] Abdel-Aleem S., Nada M.A., Sayed-Ahmed M., Hendrickson S.C., Louis J.S., Walthall H.P., Lowe J.E. (1996). Regulation of Fatty Acid Oxidation by Acetyl-CoA Generated from Glucose Utilization in Isolated Myocytes. J. Mol. Cell. Cardiol..

[16] Sara T., Sidney B., Ute S. (2015). De novo fatty acid biosynthesis and elongation in very long-chain acyl-CoA dehydrogenase-deficient mice supplemented with odd or even medium-chain fatty acids. Febs J..

[17] Raychaudhuri S., Young B.P., Espenshade P.J., Loewen C. (2012). Regulation of lipid metabolism: A tale of two yeasts. Curr. Opin. Cell Biol..

[18] Williams M.L., Menon G.K., Hanley K.P. (1992). HMG-CoA reductase inhibitors perturb fatty acid metabolism and induce peroxisomes in keratinocytes. J. Lipid Res..

[19] Brandi J., Dando I., Pozza E.D., Biondani G., Jenkins R., Elliott V., Park K., Fanelli G., Zolla L., Costello E. (2017). Proteomic analysis of pancreatic cancer stem cells: Functional role of fatty acid synthesis and mevalonate pathways. J. Proteom..

[20] Yang J., Nie Q., Liu H., Xian M., Liu H. (2016). A novel MVA-mediated pathway for isoprene production in engineered E. coli. BMC Biotechnol..

[21] He B., Tu Y., Jiang C., Zhang Z., Li Y., Zeng B. (2019). Functional Genomics of Aspergillus oryzae: Strategies and Progress. Microorganisms.

[22] Sun Y., Niu Y., Huang H., He B., Ma L., Tu Y., Tran V.-T., Zeng B., Hu Z. (2019). Mevalonate Diphosphate Decarboxylase MVD/Erg19 Is Required for Ergosterol Biosynthesis, Growth, Sporulation and Stress Tolerance in Aspergillus oryzae. Front. Microbiol..

[23] Hu Z., Li G., Sun Y., Niu Y., Ma L., He B., Ai M., Han J., Zeng B. (2018). Gene transcription profiling of Aspergillus oryzae 3.042 treated with ergosterol biosynthesis inhibitors. Braz. J. Microbiol..

[24] Liu X., Jiang J., Shao J., Yin Y., Ma Z. (2010). Gene transcription profiling of Fusarium graminearum treated with an azole fungicide tebuconazole. Appl. Microbiol. Biotechnol..

[25] Cirigliano A., Macone A., Bianchi M.M., Oliaro-Bosso S., Balliano G., Negri R., Rinaldi T. (2019). Ergosterol reduction impairs mitochondrial DNA maintenance in S. cerevisiae. BBA-Mol. Cell Biol. Lipids.

[26] Ward D.M., Chen O.S., Li L., Kaplan J., Bhuiyan S.A., Natarajan S.K., Bard M., Cox J.E. (2018). Altered sterol metabolism in budding yeast affects mitochondrial iron-sulfur (Fe-S) cluster synthesis. J. Biol. Chem..

[27] Dimmer K.S., Fritz S., Fuchs F., Messerschmitt M., Weinbach N., Neupert W., Westermann B. (2002). Genetic basis of mitochondrial function and morphology in Saccharomyces cerevisiae. Mol. Biol. Cell.

[28] Sun Y., Niu Y., He B., Ma L., Li G., Tran V.T., Zeng B., Hu Z. (2018). A dual selection marker transformation system using Agrobacterium tumefaciens for the industrial Aspergillus oryzae 3.042. J. Microbiol. Biotechnol..

[29] Long M., Li Z.Q., Lei B., Cai X.M., Luo Z.X., Zhang Y.J., Chen Z.M. (2016). Identification and Comparative Study of Chemosensory Genes Related to Host Selection by Legs Transcriptome Analysis in the Tea GeometridEctropis obliqua. PLoS ONE.

[30] Audic S., Claverie J.M. (1997). The Significance of Digital Gene Expression Profiles. Genome Res..

[31] Blanc G., Gallot-Lavallée L., Maumus F. (2015). Provirophages in the Bigelowiella genome bear testimony to past encounters with giant viruses. Proc. Natl. Acad. Sci. USA.

[32] Mannazzu I., Angelozzi D., Belviso S., Budroni M., Farris G.A., Goffrini P., Lodi T., Marzona M., Bardi L. (2008). Behaviour of Saccharomyces cerevisiae wine strains during adaptation to unfavourable conditions of fermentation on synthetic medium: Cell lipid composition, membrane integrity, viability and fermentative activity. Int. J. Food Microbiol..

[33] Ma L., Fu L., Hu Z., Li Y., Zheng X., Zhang Z., Jiang C., Zeng B. (2019). Modulation of Fatty Acid Composition of Aspergillus oryzae in Response to Ethanol Stress. Microorganisms.

