# Supplementary figures and images for: Effects on Gene Transcription Profile and Fatty Acid Composition by Genetic Modification of Mevalonate Diphosphate Decarboxylase MVD/Erg19 in Aspergillus Oryzae
Source: Microorganisms. 2019 Sep 11;7(9):342. doi: 10.3390/microorganisms7090342 (PMC6780523; doi:10.3390/microorganisms7090342)

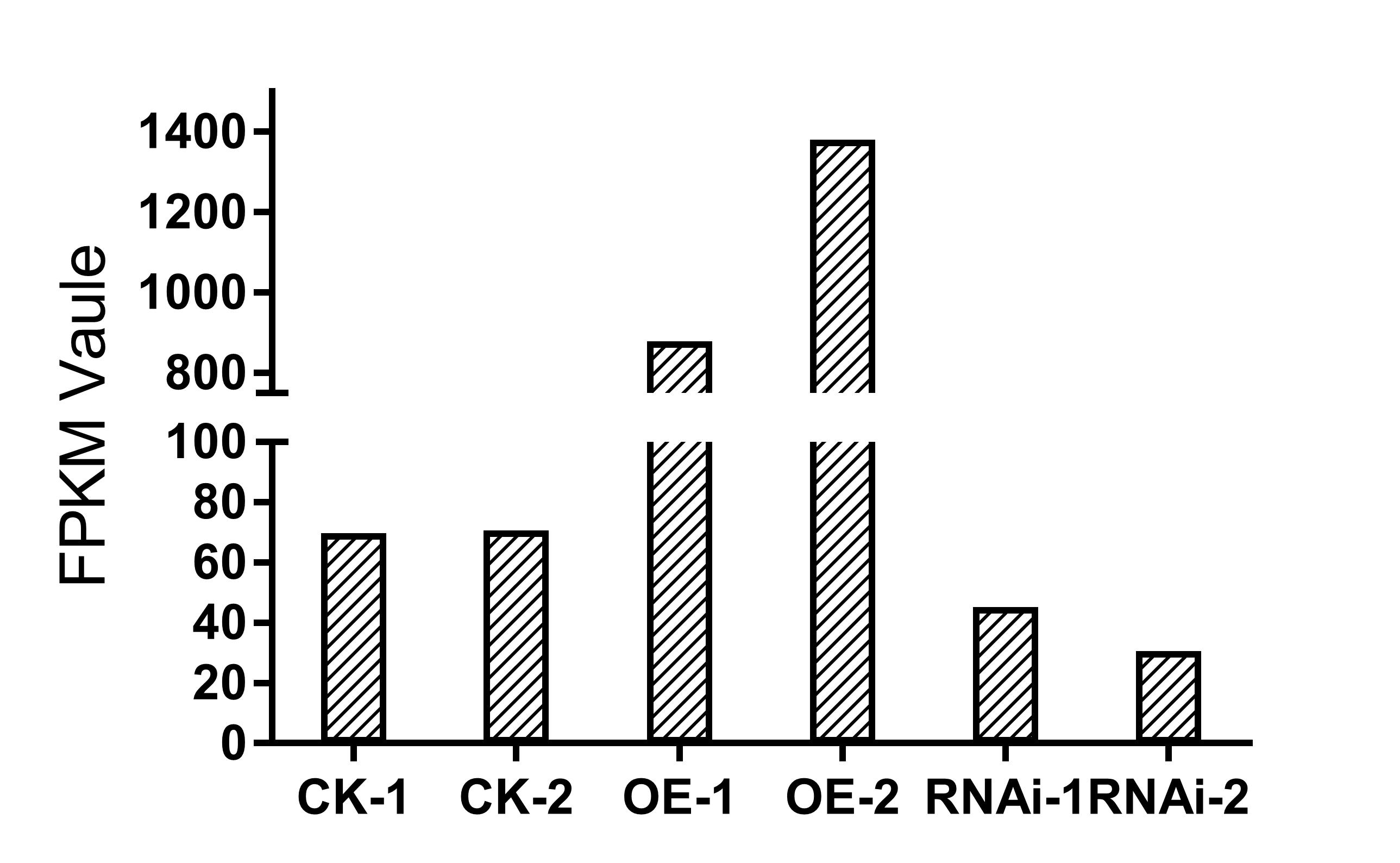

Supplement: Supplementary file 1 [file microorganisms-07-00342-s001.zip › supplementary files/Fig S1.jpg]
